# Genetic and Environmental Influences on Different Forms of Bullying Perpetration, Bullying Victimization, and Their Co-occurrence

**DOI:** 10.1007/s10519-019-09968-5

**Published:** 2019-09-10

**Authors:** Sabine A. M. Veldkamp, Dorret I. Boomsma, Eveline L. de Zeeuw, Catharina E. M. van Beijsterveldt, Meike Bartels, Conor V. Dolan, Elsje van Bergen

**Affiliations:** 1 Department of Biological Psychology, Vrije Universiteit Amsterdam, van der Boechorststraat 7, 1081 BT Amsterdam, The Netherlands; 2 Amsterdam Public Health Research (APH), Amsterdam, The Netherlands

**Keywords:** Bullying, Victimization, Bully-victims, Twins, Heritability, School

## Abstract

Bullying comes in different forms, yet most previous genetically-sensitive studies have not distinguished between them. Given the serious consequences and the high prevalence of bullying, it is remarkable that the aetiology of bullying and its different forms has been under-researched. We present the first study to investigate the genetic architecture of bullying perpetration, bullying victimization, and their co-occurrence for verbal, physical and relational bullying. Primary-school teachers rated 8215 twin children on bullying perpetration and bullying victimization. For each form of bullying, we investigated, through genetic structural equation modelling, the genetic and environmental influences on being a bully, a victim or both. 34% of the children were involved as bully, victim, or both. The correlation between being a bully and being a victim varied from 0.59 (relational) to 0.85 (physical). Heritability was ~ 70% for perpetration and ~ 65% for victimization, similar in girls and boys, yet both were somewhat lower for the relational form. Shared environmental influences were modest and more pronounced among girls. The correlation between being a bully and being a victim was explained mostly by genetic factors for verbal (~ 71%) and especially physical (~ 77%) and mostly by environmental factors for relational perpetration and victimization (~ 60%). Genes play a large role in explaining which children are at high risk of being a victim, bully, or both. For victimization this suggests an evocative gene-environment correlation: some children are at risk of being exposed to bullying, partly due to genetically influenced traits. So, genetic influences make some children more vulnerable to become a bully, victim or both.

**Electronic supplementary material:**

The online version of this article (10.1007/s10519-019-09968-5) contains supplementary material, which is available to authorized users.

## Introduction

Bullying in schools can take different forms: it can be direct (like name calling and hitting) and indirect (like social exclusion), but it always captures an element of power imbalance between the victim and the bully (or perpetrator). Involvement in bullying as a victim or bully, irrespective of the exact form, can have detrimental short- and long term effects (e.g., Nansel et al. 2004; Arseneault 2018; Kretschmer et al. 2018). Both bullies and their victims suffer for example from low self-esteem, depression, and anxiety (e.g., Kaltiala-Heino et al. 2000; Silberg et al. 2016). There are also differences between bullies and victims. For instance, bullies tend to suffer from impulsive behavior (O’Brennan et al. 2009), while victims have an increased risk of taking their own life (Gini and Espelage 2014). In addition to children who either bully or are bullied, there are children who are both bullied and bully themselves. These bully-victims suffer most from depression and anxiety (e.g., Swearer et al. 2001). Bullying is a common phenomenon (e.g., Shetgiri 2013), and it is important to understand why and how children differ with respect to this phenomenon. As for different forms, verbal and physical victimization are especially linked to aggression, while relational victimization (e.g., social exclusion or spreading rumors) is more associated with internalizing problems (Casper and Card 2017), underlining the need to study them separately. The current study explores the genetic and environmental contributions to different forms of bullying perpetration (throughout this paper termed as perpetration), bullying victimization (termed as victimization), and their co-occurrence.

Being a bully or victim tend to run in families (Allison et al. 2014; Farrington 1993). First, with respect to victimization, Allison et al. (2014) showed that a parents’ past history of victimization is associated with an increased risk of their offspring being victimized. Whereas only 25% of the parents without a past history of being bullied reported that their offspring was victimized, in the case of parents, who had been victimized themselves, this proportion was 55%. Second, with respect to perpetration, Farrington (1993) observed a comparable inter-generational continuity. Whereas only 5.5% of the fathers who did not bully had children who bullied, 16% of the fathers who were bullies reported that their children were bullies as well. Together, these family-risk studies show that perpetration and victimization are familial, but not whether this familial transmission is genetic or environmental in nature. To determine the role of genetic and shared environmental factors, we require a genetically informative design, such as the twin design.

Several twin studies have investigated the causes of individual differences in victimization, but only one investigated the causes of individual differences in perpetration and its association with victimization. The twin studies on victimization showed mixed results. Brendgen et al. (2013, 2015) found a heritability of 32% in a sample of ~ 300 6–12 year old twin pairs using teacher-reports (2013) and a heritability of 45% in ~ 200 10-year-old twin pairs using self-reports (2015). Shakoor et al. (2015) reported a similar heritability estimate of 35% in a sample of ~ 5000 12-year-old twin pairs using self-reports. Silberg et al. (2016) studied ~ 1400 8–17 year old twin pairs using mother and child self-reports (combined) and reported a heritability estimate of 45%. In contrast, Ball et al. (2008) reported a higher heritability estimate of 73% in a sample of ~ 1100 10-year-old twin pairs using mother-reports. Connolly and Beaver (2016) found a heritability of 70% in a sample of ~ 300 12–16 year old twin pairs, who reported their history of suffering repeated bullying before age 12. Bowes et al. (2013) showed that in a sample of ~ 1100 twin pairs the heritability of victimization (mother and self-reports combined) was 71% in primary school and 77% in secondary school. The diverging results may be due to differences in informant (e.g., self vs. parental report), age of the participants, and (or) the type of assessment.

Moving on to perpetration, the only twin-study reported a heritability of 61% at age 10 (Ball et al. 2008). Ball et al. were also the only ones that tested whether the genetic and environmental influences on both victimization and perpetration differed in boys and girls, and found no difference. Moreover, Ball et al. (2008) looked at the co-occurrence of perpetration and victimization, which correlated 0.25. This correlation was found to be solely due to genetic factors common to perpetration and victimization.

The twin-studies mentioned above did not differentiate between various types of bullying, but Eastman et al. (2018) recently investigated for the first time genetic and environmental influences on different forms of victimization. The heritability estimates of self-reported verbal, physical, relational, and property victimization in early adolescence ranged from 23% for attacks on property to 42% for physical victimization. Due to limited power (*N *= 306 pairs in the youngest of two age groups) they could not investigate whether heritability differed between boys and girls. Perpetration was not investigated.

We know that gender and the form of bullying influence prevalence rates. Specifically, most studies report that boys are more likely to be involved in bullying than girls, either as bully or victim (e.g. Nansel et al. 2001; Veldkamp et al. 2017). However, the form of bullying has a bearing on these gender-differences (e.g. Crick and Nelson 2002; Cullerton-Sen and Crick 2005). Boys are more often involved in verbal (e.g. name-calling) and physical bullying (e.g. hitting), while girls are more involved in relational bullying (e.g. social exclusion). Importantly, it remains to be investigated whether genetic and environmental influences differ in boys and girls and differ between the forms of bullying. The present study is the first to investigate the genetic and environmental influences on general, verbal, physical, and relational perpetration and victimization, and on the covariance between them.

## Method

### Participants

Primary school teachers provided information concerning perpetration and victimization in 8215 twins from 4561 pairs. Of these pairs, 1669 were MZ (monozygotic) and 2892 were DZ (dizygotic; 53% of the DZ twin pairs were of same sex and 47% were of opposite sex). The twins were enrolled in the Netherlands Twin Register (NTR; Van Beijsterveldt et al. 2013), which was established by the Department of Biological Psychology at the Vrije Universiteit Amsterdam. The project was approved by the medical ethical committee of the Vrije Universiteit Amsterdam (NTR/25-05-2007). Parents of the twins, aged 7, 9, and 12 years, provided their consent to approach the teachers of the twins with a survey. Since 2010, the survey for the primary school teachers has included four items on perpetration and four items on victimization. The current study is a follow-up study of Veldkamp et al. (2017), that focused on the prevalence of perpetration and victimization, and included the same data, which were collected between 2010 and 2015. Data were excluded if (1) zygosity was unknown (*N *= 193), (2) the teacher was not sufficiently familiar with the child (*N *= 74), (3) the child was rated by someone other than the regular teacher (*N *= 81), (4) the twins were in separate classrooms, but rated by the same teacher (*N *= 11), (5) the twins were in the same classroom but rated by different teachers (*N *= 108). The 8215 twin children in the final dataset had data for at least one wave.

The data are characterized by a small degree of dependency. A subset of children had data on two (*N *= 1617) or three (*N *= 93) time points, resulting in a total sample of 10,018 observations. We conducted the analyses with the complete data recognizing that the dependency may bias-down the standard errors. After removing the dependent cases and rerunning the analyses differences in the results were trivial. We also reran the analyses with the Mplus complex option, which corrects standard errors for the dependency. Again the differences in standard errors were trivial. Given lack of appreciable differences we proceeded with the original results.

Of the MZ twins, 45.1% attended separate classrooms and 54.9% the same. In the DZ twins, these figures were 49.5% and 50.5%, respectively. Incomplete data (*N *= 1384 twin pairs) was mostly due to one of the teachers not returning the survey when the twins attended separate classrooms (*N *= 1232). The age of the children ranged from 6.52 to 12.94 years (*M *= 9.48, *SD *= 2.01). The degree of perpetration and victimization did hardly change with age, as indicated by correlations between age and the eight phenotypes, which ranged from − 0.12 to 0.07. Hence, age effects were not further investigated.

### Measures

#### Perpetration and victimization

Teachers received a survey which included four items about perpetration and four matched items about victimization. Each item concerns general, physical, verbal, or relational perpetration and victimization. The 2 × 4 questions were scored on a five-point response scale, ranging from 0 (*never*), 1 (*once or twice*), 2 (*two or three times a month*), 3 (*about once a week*), to 4 (*several times a week*). The four items for bullying victimization were: ‘How often has this student in the last couple of months… a) been bullied (in general), b) been teased, laughed at, or called names? (verbal), c) been physically bullied, such as being hit, kicked, and pushed? (physical), d) been excluded by other children, ignored, or have other students spread false rumors? (relational)’. The parts between brackets (e.g., “relational”) were indeed part of the teacher items. For the original Dutch items, see the Supplementary Materials Online. Bullying perpetration was assessed with the same items, but formulated to reflect the active form (e.g. ‘How often did this student in the last couple of months… a) bully other students (in general)’). Missingness at the level of the individual items was less than 1.6%.

In the case of general, verbal, and relational perpetration and victimization items, the last two response options (i.e. “*about once a week*” and “*several times a week*”) were rarely chosen. Similarly, the last three response options of the physical perpetration and victimization items were rarely chosen. We therefore transformed the response scale of the general, verbal and relational items to three categories, and the response scale of the physical items to two categories.

### Statistical analyses

First, we present the prevalence of being involved in the various types of perpetration and victimization, and the phenotypic correlations. Next, we present the results of the analyses of the twin data using genetic structural equation modeling. These results include the decomposition of the phenotypic bivariate covariance matrix (perpetration–victimization) into genetic and environmental components.

#### Behavioral genetic analyses plan

**Fig. 1:**
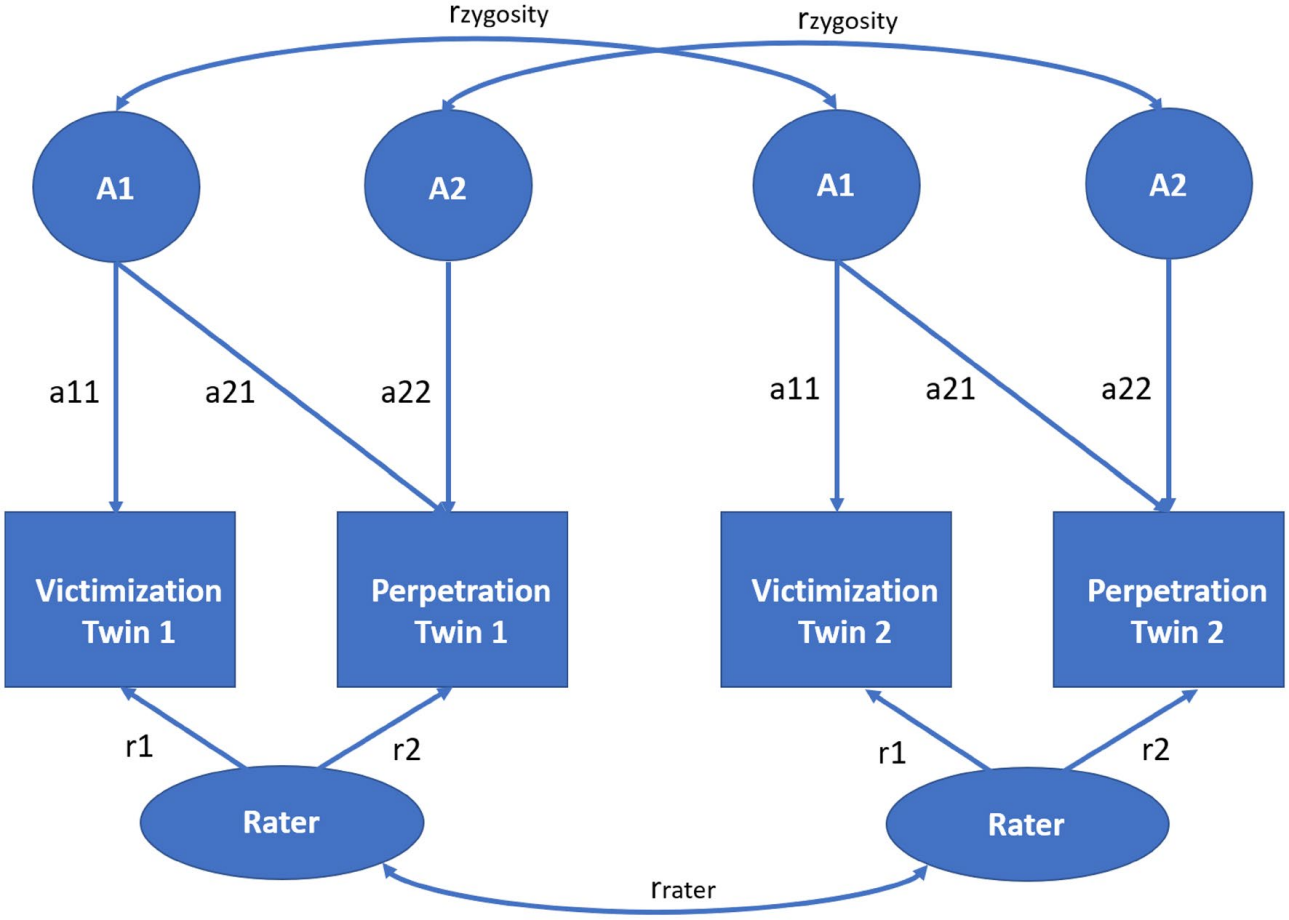
Bivariate Cholesky ACE decomposition including rater bias. “A” represents the genetic influences. The common environmental (C) and unique environmental (E) influences are not shown to avoid clutter (but can be found in Fig. S1 in the Supplementary Materials). “r_zygosity_” is 1 for MZ twins and 0.5 for DZ twins. “r_rater_” represents the correlation between the raters of the twin, which is 1 for twins rated by the same teacher and 0 for twins rated by different teachers. “a11” represents the genetic influences on victimization, “a12” represents the genetic covariance between victimization and perpetration, and “a22” represents the unique genetic influences on perpetration after accounting for the shared genetic influences. This model was fitted to each type of perpetration/victimization pair

In twin studies, we use the ACE model to decompose phenotypic variances and covariances into genetic, common and unique environmental variance components. The A (in ACE) represents additive genetic influences, the C represents environmental influences that are shared by siblings (i.e., common) and lead to similarities between them, and E represents unique environmental influences, which make siblings less alike, and measurement error. The decomposition is based on the fact that MZ twins are genetically identical, while DZ twins on average share 50% of the alleles that make up segregating genes. Consequently, if the MZ twin correlation is larger than the DZ twin correlation, this suggests genetic influences. If twice the DZ correlation is greater than the MZ correlation, this suggests shared environmental influences (C). In contrast, if twice the DZ correlation is smaller than the MZ correlations, this suggests dominance influences (D). MZ twin correlations are invariably less than one, which imply the presence of unshared or unique environmental influences and measurement error (together E), which contribute to twin differences. In a twin study, C and D cannot be estimated simultaneously, so based on the twin correlations, either an ACE or ADE model is fitted. In practice the decomposition is carried out by fitting the ACE model (or ADE model) to the twin data using genetic structural equation modeling (Posthuma et al. 2003). This allows us to generalize the decomposition to multiple phenotypes. In the present case, we decompose the phenotypic 2 × 2 covariance matrix (perpetration–victimization by type of bullying) into 2 × 2 A, C and E covariance matrices. This provides information on the contributions of genetic and environmental factors to the variance of the phenotypes and to the covariance between the phenotypes. We used the bivariate Cholesky model to obtain the bivariate decomposition. This is depicted in Fig. 1.

We assumed that raters may introduce systematic variation into the phenotype ratings, which reflect for example differences in raters’ visions of bullying. In addition, raters who assess multiple children, can cause possible rater contrast effects. More specifically, the twins in our dataset that attend the same classroom were assessed by the same teacher. This might result in more similar bullying estimates than when the twin children were assessed by different teachers, here termed as rater effects (Bartels et al. 2007; Rietveld et al. 2003a). As shown in Fig. 1, we included in the model a rater effect to accommodate this variation. The rater effects are assumed to contribute to the covariance between phenotypes within twin members. If the twins are rated by the same teacher (i.e., twins in the same class), the rater effect may also contribute to the phenotypic covariance between twins.

We used Mplus version 7 to fit the ACE twin model (Muthén and Muthén 1998–2012). As the data are ordinal, we used robust weighted least squared (WLSMV) estimation applied to the tetrachoric or polychoric correlation matrices. This is consistent with the liability-threshold modeling (e.g., Rijsdijk and Sham 2002), in which the ordinal data arise from the discretization of bivariate normal (latent) liabilities. The phenotypic summary statistics are the thresholds and the tetrachoric or polychoric correlation matrices. The correlations convey the linear association at the level of the liabilities, and the thresholds convey the frequencies of the responses. The model included 5 × 2 groups. First, five groups were based on zygosity and sex (MZ males, DZ males, MZ females, DZ females and DZ opposite-sex pairs). Given the five groups, we could test sex differences in the variance components and the thresholds. Second, each group was further divided into “same-class” and “different-class” groups (hence the 5 × 2 groups). The latter subdivision was made to accommodate the rater effects (see Fig. 1), which are shared by twins in the same class (and so the same teacher rater).

In sum, in the full model the bivariate phenotypic covariance matrix was decomposed into ACE components and the rater-variance component. The 10-group model allowed us to use χ^2^ difference testing to study sex differences in thresholds and variance components, while accounting for the rater effect. We carried out the bivariate analyses (perpetration–victimization) separately for each form of bullying (general, verbal, physical and relational).

We tested the sex and classroom effects on the genetic and environmental variance components (these tests are also represented in Table S1 in the Supplementary Materials Online). First, we tested whether the thresholds (i.e., the prevalences) depended on class sharing and gender. Second, we tested whether the ACE components varied with classroom sharing and with gender. Because twin correlations (Tables S2–S5) were consistent with an ACE pattern rather than an ADE pattern, dominance effects (D) were not investigated. This is also consistent with the previous literature on bullying that found C but no D influences (e.g. Ball et al. 2008; Connolly and Beaver 2016). The variances of MZ and DZ twins were similar, and hence we did not consider social interaction effects between twins (Eaves 1976; Rietveld et al. 2003b).

Model fit evaluation was based on the Chi squared test, the Comparative Fit Index (CFI) and the Root Mean Square Error of Approximation (RMSEA) (Kline 2011). The Chi squared test is based on the difference between the observed and expected covariance matrices, with a better fit indicated by Chi square values closer to zero. Model evaluation was based on the combinational rule of Chi squared p-values > 0.05, CFI > 0.95, and RMSEA < 0.05. Comparison of a model with a reduced model was based on χ^2^ difference testing. To accommodate multiple testing, we used an adjusted α-value of 0.01. The data were prepared in R, version 3.4.1 (R Core Team 2017) and all models were fitted in Mplus, version 7 (Muthén and Muthén 1998–2012).

## Results

### Descriptive statistics

The prevalences of children involved in perpetration and/or victimization are shown in Table 1. For these prevalence rates the response categories were dichotomized, with children scoring 0 (“never”) categorized as “not involved”, and children scoring 1 to 4 categorized as “involved”. Based on the item on bullying in general in Table 1, we can summarize that, according to teachers, 34% of the children had been involved in bullying over the past couple of months (as victim, bully, or both). More specifically, 8.4% was a pure victim, 11.1% a pure bully and 14.4% a bully-victim, resulting in 33.9% of the children being involved.

**Table 1:** Prevalence of victimization and perpetration by sex

	Total sample (%)	Sex	
		Boys (%)	Girls (%)
Percentage victims^a^			
General	23	27	19
Verbal	25	30	19
Physical	8	11	4
Relational	17	15	21
Percentage bullies^a^			
General	26	34	17
Verbal	26	36	17
Physical	9	15	3
Relational	20	18	22
Percentage bully-victims			
General	14	19	10
Verbal	16	21	10
Physical	5	8	2
Relational	10	8	13

The percentages include children who were involved at least once or twice in the last couple of months

^a^Including bully-victims

Boys were more often involved in bullying, either as victim or bully. Irrespective of gender, verbal bullying was most and physical bullying was least prevalent. Regarding gender and the form of bullying, boys were more involved in verbal and physical bullying (as a bully and victim), while girls were more often involved in relational bullying (as a bully and victim).

Phenotypic correlations between all forms of perpetration and victimization are represented in Table 2, separately for boys and girls. The correlations between perpetration and victimization for the same form of bullying were for boys 0.64, 0.65, 0.80, and 0.59 for general, verbal, physical and relational, respectively, and for girls 0.68, 0.72, 0.85, and 0.68.

**Table 2:** Correlations between various forms of perpetration and victimization by sex

	Victimization				Perpetration			
	General	Verbal	Physical	Relational	General	Verbal	Physical	Relational
Victimization								
General	–	0.85	0.60	0.76	**0.68**	0.56	0.53	0.47
Verbal	0.88	–	0.61	0.70	0.59	**0.72**	0.59	0.50
Physical	0.68	0.65	–	0.47	0.49	0.52	**0.85**	0.36
Relational	0.75	0.71	0.53	–	0.54	0.52	0.47	**0.68**
Perpetration								
General	**0.64**	0.56	0.56	0.47	–	0.86	0.68	0.83
Verbal	0.54	**0.65**	0.56	0.49	0.90	–	0.69	0.78
Physical	0.51	0.51	**0.80**	0.48	0.73	0.71	–	0.51
Relational	0.35	0.41	0.44	**0.59**	0.75	0.77	0.56	–

Correlations are shown above the diagonal for girls and below the diagonal for boys. Correlations between the same form of perpetration and victimization are shown in bold typeface

The model estimated twin correlations for all items are shown in Tables S2–S5. For all items the MZ correlation was higher than the DZ correlation, indicating genetic influences. The cross-twin cross-trait correlations were also all higher for MZ twins than DZ twins, suggesting that genes contribute to the perpetration-victimization association.

### Bivariate genetic modeling

For each form of bullying, the same model fitting procedure was followed. A summary of the statistical details of the model fitting steps for the model of the general item can be found in Table 3. The model fitting steps of the model for this general item and for the models of the other items are described in more detail in the Supplementary Materials Online.

**Table 3:** Summary of the modelling steps for the general item

Equality constraints	Model fit						Model comparison^a^			
	*Ep*	χ^2^	*df*	*p* value	CFI	RMSEA	Models	∆χ^2^	∆*df*	*p*-value
0 saturated model	46	123.66	94	0.022	0.997	0.024	–	–	–	–
1 ACE: same means in same class and different classes	38	161.14	102	< 0.001	0.994	0.032	1 vs. 0	32.83	8	< 0.001
2 ACE: same means in boys and girls	38	678.37	102	< 0.001	0.942	0.100	2 vs. 0	470.74	8	< 0.001
3 ACE: no classroom differences in all genetic parameters	40	127.78	100	0.032	0.997	0.022	3 vs. 0	5.25	6	0.512
4 ACE: no classroom differences in all common environmental parameters	34	139.27	106	0.017	0.997	0.024	4 vs. 3	11.52	6	0.074
5 ACE: no classroom differences in unique environmental covariation	32	140.63	108	0.019	0.997	0.023	5 vs. 4	2.03	2	0.363
6 ACE: no sex differences in all genetic parameters	**29**	**144.99**	**111**	**0.017**	**0.997**	**0.023**	**6 vs. 5**	**5.26**	**3**	**0.154**
7 ACE: no sex differences in all common environmental parameters and in unique environmental covariation	25	167.19	115	0.001	0.995	0.028	7 vs. 6	21.33	4	< 0.001

Each model was compared with the previous best-fitting model. The best-fitting model is shown in bold. This model has equal influences of genetic, common-, and unique environmental factors for twins in the same and separate classrooms. For boys and girls, the influence of genetic factors was the same, but the influence of common and unique environmental factors differed

*Ep* estimated parameters, *df* degrees of freedom

^a^The Chi square values of the models themselves cannot be used for Chi square differences testing in the regular way, since the WLSMV estimator in Mplus was used. The results of the Chi square difference test was based on the “difftest” option in Mplus

**Fig. 2:**
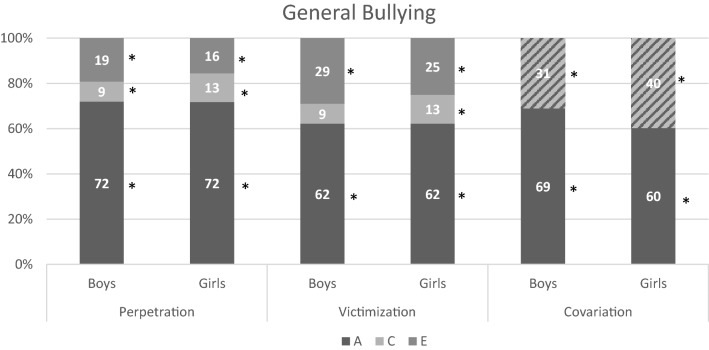
Results for general bullying for boys/girls. The covariation is divided into shared effects (A) and environmental effects (C + E). Note that * indicates significance (based on 95% confidence intervals)

**Fig. 3:**
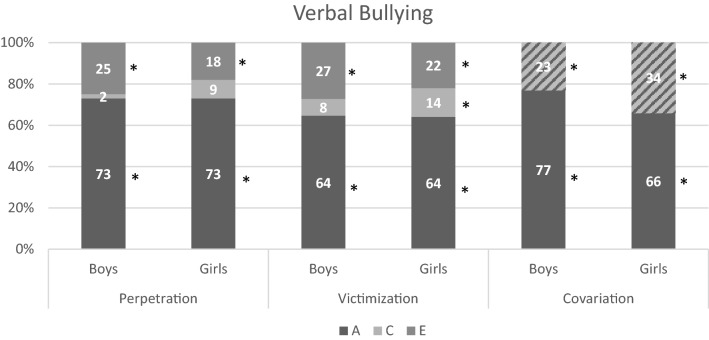
Results for verbal bullying for boys/girls. The covariation is divided into shared effects (A) and environmental effects (C + E). Note that * indicates significance (based on 95% confidence intervals)

**Fig. 4:**
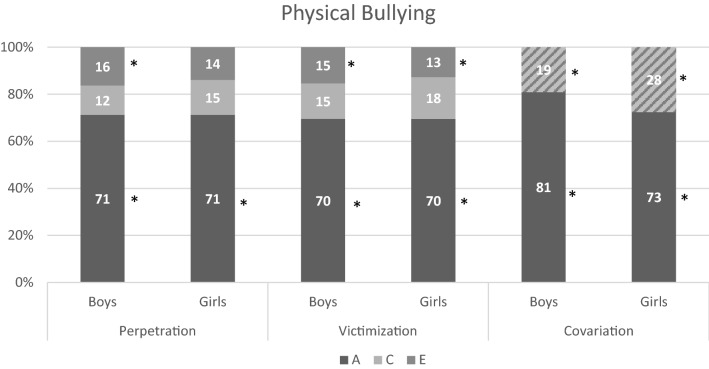
Results for physical bullying for boys/girls. The covariation is divided into shared effects (A) and environmental effects (C + E). Note that * indicates significance (based on 95% confidence intervals)

**Fig. 5:**
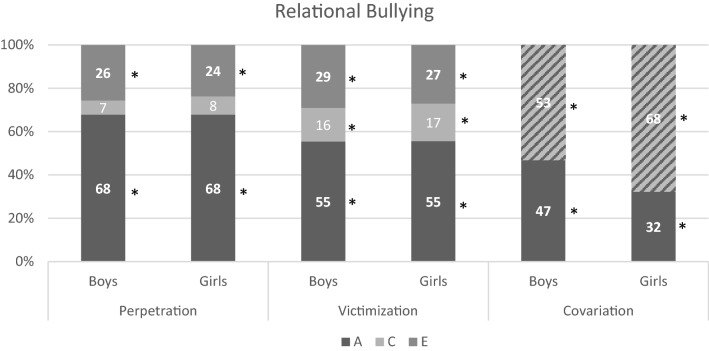
Results for relational bullying for boys/girls. The covariation is divided into shared effects (A) and environmental effects (C + E). Note that * indicates significance (based on 95% confidence intervals)

For all forms the best fitting model was an ACE model with equal influences of genetic, common-, and unique environmental factors for twins in the same and separate classrooms. For boys and girls, the influence of genetic factors was the same, but the influence of common and unique environmental factors differed. The standardized estimates for variation due to additive genetic (A), common environmental (C) and unique environmental (E) factors, and rater estimates are shown in Table S6 and the estimates after accounting for rater effects in Table 4. Summaries of the results are visualized in Figs. 2, 3, 4, and 5. All forms of bullying showed substantial genetic influences. General perpetration and general, verbal, and relational victimization showed small shared environmental influences, which were more often significant in girls. The association between perpetration and victimization was for most forms mainly genetic in nature.

**Table 4:** Estimates (in  %) for variation due to additive genetic, common environmental, and unique environmental factors for all types of perpetration, victimization, and their correlation, after accounting for the rater-effects, with 95% confidence intervals between brackets

	Perpetration						Victimization						Correlation			
	A		C		E		A		C		E		*r*_A_		Proportion due to A^a^	
	Boys	Girls	Boys	Girls	Boys	Girls	Boys	Girls	Boys	Girls	Boys	Girls	Boys	Girls	Boys	Girls
General	72^(64–80)^		9^(1–16)^	13^(4–21)^	19^(15–24)^	16^(10–21)^	62^(52–73)^		9^(0–18)^	13^(3–23)^	29^(22–37)^	25^(17–34)^	0.50^(0.37–0.62)^		69^(52–87)^	60^(44–77)^
Verbal	73^(60–86)^		2^(−9 to 14)^	9^(−4 to 22)^	25^(19–30)^	18^(12–24)^	64^(55–74)^		8^(0–17)^	14^(4–23)^	27^(21–34)^	22^(14–30)^	0.62^(0.48–0.77)^		77^(63–91)^	66^(53–79)^
Physical	71^(54–89)^		12^(−4 to 29)^	15^(−4 to 34)^	16^(9–24)^	14^(0–28)^	70^(52–87)^		15^(−1 to 31)^	18^(0–36)^	15^(6–25)^	13^(9–25)^	0.86^(0.72–1.00)^		81^(62–100)^	73^(56–90)^
Relational	68^(57–79)^		7^(−4 to 17)^	8^(−2 to 19)^	26^(18–33)^	24^(17–31)^	55^(42–69)^		16^(4–28)^	17^(6–29)^	29^(18–40)^	27^(19–36)^	0.26^(0.05–0.47)^		47^(11–83)^	32^(6–59)^

Estimates for heritability were constrained to be equal for boys and girls. The rater effect was also set equal for boys and girls

^a^The proportion of the correlation (between perpetration and victimization) that is due to A differs between boys and girls, because the phenotypic correlations between perpetration and victimization differed

## Discussion

In a sample of 8215 primary-school children, we showed that individual differences in the liability to be a victim, bully, or bully-victim are mainly due to genetic differences between children. We asked teachers to give their view of general, verbal, physical, and relational bullying. After accounting for rater effects (twins rated by the same or different teachers), the genetic influences for both boys and girls were high for all forms of perpetration (~ 70%), and for general-, verbal-, and physical victimization (~ 65%), but somewhat lower for relational victimization (55%). The correlation between bully and victim roles was ~ 0.70. This correlation was mostly due to shared genetic factors for the verbal and physical form and mostly due to an overlap in (common and unique) environmental factors for the relational form.

Teachers reported that the proportion of children that had been involved in bullying over the past couple of months (either as bully, victim, or both) was one-third. We showed that, irrespective of gender and role (bully, victim, bully-victim), physical bullying was least prevalent and verbal bullying was most prevalent. Verbal and especially physical bullying was more common in boys, while relational bullying was more observed among girls. These prevalences provide a background for interpreting the etiological findings below.

Regarding victimization, two-thirds of the phenotypic variance expressing individual differences was due to genetic influences. At first sight, it may seem odd to claim that victimization is highly heritable, since it is an exposure to a school environment in which the child is bullied rather than direct behavior. The heritability can, however, be explained by other heritable traits that increase victimization risk. For instance, internalizing problem behavior and low self-esteem put children at greater risk to become a victim (Tsaousis 2016) and these traits themselves are moderately heritable (Bartels et al. 2004). In addition, the risk of victimization increases with increased BMI (Janssen et al. 2004), which is highly heritable (Nan et al. 2012). Consequently, these genetically influenced traits might elicit harsh treatment by peers, leading to an evocative gene-environment correlation.

Regarding perpetration, around 70% of the individual differences were caused by genetic factors. This is slightly more than the 61% that was found in the only previous study (Ball et al. 2008). The heritability of perpetration might be easier to understand, since it is direct behavior rather than an exposure. Our finding of a heritability of ~ 70% for bullying perpetration is in line with the moderate heritability estimates of the related traits antisocial behavior (Rhee and Waldman 2002) and aggression (Hudziak et al. 2003). Bullying perpetration is one element of antisocial behavior and aggression, as reflected by questionnaires on these traits which typically include an item on bullying perpetration. For example, the aggression scale of the Child Behavior Checklist (CBCL; Achenbach and Rescorla 2001) includes the item “*Cruelty, bullying or meanness to others*”. It has previously been suggested that genetically influenced traits such as impulsivity could mediate the genetic effects of antisocial behavior (Jacobson et al. 2002), and this might also apply to perpetration. Bullies have indeed higher levels of impulsivity (O’Brennan et al. 2009).

Being a bully or victim of physical bullying is, compared to the other subtypes, to a lesser extent affected by unique environmental factors. Unique environmental factors include factors not shared in a twin pair, as well as measurement error. Measurement error could be reduced because physical bullying is more visible for teachers than, for instance, relational bullying. Conversely, relational bullying being least heritable might be partly due to more measurement error. In accordance with this idea, Eastman et al. (2018) also showed that physical victimization is most heritable.

For all forms of bullying (both perpetration and victimization), the influence of the common environment was modest and was slightly higher for girls than for boys. About half of the common-environment estimates reached statistical significance. This is in line previous mixed results: Ball et al. (2008) did not find significant influences of the common environment on perpetration and victimization, but Brendgen et al. (2008) found a significant influence on victimization. Our finding of a significant influence of the common environment on general perpetration is in line with a common-environmental influence on the related phenotypes aggression and antisocial behavior (Miles and Carey 1997). The slightly higher influence of the common environment for girls indicates that the school and/or home environment are more important for girls. To illustrate, pairs of sisters are closer than other pairs of siblings (Buist 2010).

The co-occurrence of perpetration and victimization, reflecting bully-victims, was mainly due to genetic factors for verbal and physical bullying, but mainly due to environmental factors for relational bullying. Ball et al. (2008), the only study done so far, showed that the phenotypic correlation between perpetration and victimization was low (0.25) and mostly due to genetic factors. Here we demonstrate that the influences on the co-occurrence depend on type of bullying. The genetic influences on the co-occurrence might be explained in two ways. First, it might be that the same genes influence both phenotypes via another heritable characteristic, like aggression. Bully-victims are the most aggressive group, compared to ‘pure’ bullies and victims (Salmivalli and Nieminen 2002). Their genetic liability for aggression makes them more likely to get involved in a fight without necessarily a clear role as a bully or victim. Second, there might be phenotypic causality, meaning that being a bully (a genetically-mediated trait) makes a child less popular and therefore more vulnerable to become a victim as well (or vice versa). Indeed, bully-victims are the most disliked group (Veenstra et al. 2005).

In interpreting these results, it is important to mention that our results are based on teacher ratings and that phenotypes are based on only one item each. In general, teacher ratings are not highly correlated with parent and self-ratings. For perpetration, Ball et al. (2008) found a modest correlation between teacher and mother reports (*r *= 0.24). Our results may therefore present situation-specific prevalences and etiology, meaning that other influences might be responsible for school-bullying than for bullying that happens out of the sight of the teacher. For aggression, however, disagreement between teacher and mother ratings did not cause different heritability estimates (Hudziak et al. 2003). The strengths of our study include: (1) our large sample and genetically-informative design, (2) investigating subtypes of perpetration and victimization measured in the same way, (3) estimating effects free of rater effects (which was for different forms of perpetration 17–37% and for victimization 34–43%).

Some children are at risk of being exposed to bullying, partly due to genetically influenced traits, but this does not mean that bullying behavior is not modifiable. Those who work with children know that children who are outliers in some ways (e.g. behavior and appearance) are more vulnerable (Arseneault 2018). Behavior and physical appearance are moderately to highly genetically influenced (Polderman et al. 2015). Still, bullying in schools can be reduced by creating supportive environments with evidence-based interventions (Gaffney et al. 2018).

To conclude, this study is a first step to identify why some children are involved in different types of bullying and others are not. Our results revealed that both perpetration and victimization are substantially heritable, and that their co-occurrence is mostly due to shared genetic influences for verbal and physical bullying, but mostly due to an overlap in environmental influences for relational bullying. It must be stressed that it is certainly not someone’s fate to be a bully or victim (or both), but some children are more vulnerable to these social roles, and individual differences in this vulnerability are substantially due to genetic differences. Thus, becoming a victim, bully or bully-victim is not fixed beforehand, but is not randomly determined either.

## Electronic supplementary material

Below is the link to the electronic supplementary material.

Supplementary material 1 (PDF 237 kb)

## Abbreviations

MZ: Monozygotic
DZ: Dizygotic
NTR: Netherlands Twin Register
