# Supplementary material for: Genetic and Environmental Influences on Different Forms of Bullying Perpetration, Bullying Victimization, and Their Co-occurrence
Source: Behav Genet. 2019 Sep 10;49(5):432–43. doi: 10.1007/s10519-019-09968-5 (PMC6768918; doi:10.1007/s10519-019-09968-5)
Supplement: Supplementary file 1 — Supplementary material 1 (PDF 237 kb) [file 10519_2019_9968_MOESM1_ESM.pdf]

Supplementary material for "*Genetic and environmental influences on different form of bullying perpetration, bullying victimization and their co-occurrence*" in Behavior Genetics.

Please find here the online version of the article: https://doi.org/10.1007/s10519-019-09968-5

Sabine A.M. Veldkamp[1,2], Dorret I. Boomsma[1,2], Eveline L. de Zeeuw[1,2], Catharina E.M. van Beijsterveldt[1], Meike Bartels[1,2] , Conor V. Dolan[1,2], Elsje van Bergen[1,2*]

[1] Department of Biological Psychology, Vrije Universiteit Amsterdam, the Netherlands
[2] Amsterdam Public Health Research (APH), Amsterdam, the Netherlands
* Email: e.van.bergen@vu.nl; Twitter: @drElsje

Detailed model fitting steps

For each form of perpetration and victimization, the same model fitting procedure was followed. Results of this model fitting procedure are given apart for each item.

General perpetration-victimization. The full bivariate model gave an excellent fit to the data (χ^2 (94, N = 5,634 twin pairs) = 123.66, p = .022 *CFI* = .997, *RMSEA* = .024 (90% Confidence Interval (CI): .010 - .034)). The thresholds could not be constrained to be equal for twins in same and separate classrooms ($\Delta\chi^2$(8, N = 5,634 twin pairs) = 32.83, $p < .001$) nor for boys and girls ($\Delta\chi^2$(8, N = 5,634 twin pairs) = 470.74, $p < .001$) and were therefore freely estimated in subsequent models. For twins in the same and separate classrooms, the genetic parameters ($\Delta\chi^2$(6, N = 5,634 twin pairs) = 5.25, p = .512), the common environment parameters ($\Delta\chi^2$(6, N = 5,634 twin pairs) = 11.52, p = .074) and the unique environment parameters could be constrained to be equal ($\Delta\chi^2$(2, N = 5,634 twin pairs) = 2.03, p = .363). The genetic influences did not differ significantly between for boys and girls ($\Delta\chi^2$(3, N = 5,634 twin pairs) = 5.26, p =

.154), but the environmental influences (both common and unique) did ($\Delta\chi^2$(4, *N* = 5,634 twin families) = 21.33, $p < .001$). In Figure 2 of the manuscript a summary of the results is visualized.

Verbal perpetration-victimization. The full bivariate model gave a satisfactory fit to the data (χ^2(94, *N* = 5,610 twin pairs) = 156.94, $p < .001$, *CFI* = .993, *RMSEA* = .035 (90% CI: .025 - .034)). The thresholds could not be constrained to be equal for twins in same and different classrooms ($\Delta\chi^2$(8, *N* = 5,610 twin pairs) = 30.18, $p < .001$) and for boys and girls ($\Delta\chi^2$(8, *N* = 5,610 twin pairs) = 621.85, $p < .001$) and are therefore freely estimated in subsequent models. For twins in the same and separate classrooms, the genetic parameters ($\Delta\chi^2$(6, *N* = 5,610 twin pairs) = 2.21, $p = .899$), the common environment parameters ($\Delta\chi^2$(6, *N* = 5,610 twin pairs) = 12.95, $p = .044$) and the unique environment parameters could be constrained to be equal ($\Delta\chi^2$(2, *N* = 5,610 twin pairs) = 2.81, $p = .246$). For boys and girls, the genetic can be constrained ($\Delta\chi^2$(3, *N* = 5,610 twin pairs) = .97, $p = .810$), but not the environmental influences ($\Delta\chi^2$(4, *N* = 5,610 twin pairs) = 21.55, $p < .001$). In Figure 3 of the manuscript a summary of the results is visualized.

Physical perpetration-victimization. The full bivariate model gave an excellent fit to the data (χ^2(62, *N* = 5,610 twin pairs) = 59.26, $p = .575$ *CFI* = 1.00, *RMSEA* = .000 (90% CI: .000 - .023)). The thresholds could be constrained to be equal for twins in same and separate classrooms ($\Delta\chi^2$(4, *N* = 5,610 twin pairs) = 2.85, $p = .583$), but not for boys and girls ($\Delta\chi^2$(2, *N* = 5,610 twin pairs) = 412.38, $p < .001$). Therefore, the thresholds for twin in the same and separate classrooms were set equal and only the thresholds for boys and girls were freely estimated in subsequent models. For twins in the same and separate classrooms, the genetic parameters ($\Delta\chi^2$(6, *N* = 5,610 twin pairs) = 2.88, $p = .824$), the common environmental parameters ($\Delta\chi^2$(6, *N* = 5,610 twin pairs) = 4.38, $p = .625$), and the unique environmental parameters ($\Delta\chi^2$(2, *N* = 5,610 twin pairs) = 7.81, $p = .020$) could be constrained to be equal. For boys and girls, the genetic parameters could be constrained to be equal ($\Delta\chi^2$(3, *N* = 5,610 twin

pairs) = 3.28, p = .351), but not the environmental (both common and unique) parameters ($\Delta\chi^2$(4, N = 5,610 twin pairs) = 13.49, p = .009). In Figure 4 of the manuscript a summary of the results is visualized.

Relational perpetration-victimization. The full bivariate model gave an excellent fit to the data (χ^2(94, N = 5,611 twin pairs) = 117.59, p = .050 *CFI* = .997, *RMSEA* = .021 (90% CI: .000 - .032)). The thresholds could not be set equal for twins in same and different classrooms ($\Delta\chi^2$(8, N = 5,611 twin pairs) = 42.43, p < .001) and for boys and girls ($\Delta\chi^2$(8, N = 5,611 twin families) = 73.65, p < .001) and were therefore freely estimated in the subsequent models. The genetic influences could be constrained to be equal for twins in the same versus separate classrooms ($\Delta\chi^2$(6, N = 5,611 twin pairs) = 12.09, p = .060), as well as the common environmental effects ($\Delta\chi^2$(6, N = 5,611 twin pairs) = 4.15, p = .657) and the unique environmental effects ($\Delta\chi^2$(2, N = 5,611 twin pairs) = 7.81, p = .020). For boys and girls, the genetic factors could be constrained to be equal ($\Delta\chi^2$(3, N = 5,611 twin pairs) = 1.85, p = .605), and the environmental factors could not ($\Delta\chi^2$(4, N = 5,611 twin pairs) = 27.29, p < .001). In Figure 5 of the manuscript a summary of the results is visualized.

Dutch items Bullying Perpetration and Bullying Victimization

Hoe vaak is deze leerling in de afgelopen maanden…

		niet	1 of 2 keer	2 of 3 keer per maand	ongeveer 1 keer per week	meerdere keren per week
a.	gepest (algemeen)	☐1	☐2	☐3	☐4	☐5
b.	gepest door belediging, uitschelden, of uitlachen? (verbaal)	☐1	☐2	☐3	☐4	☐5
c.	gepest door spugen, slaan, schoppen of knijpen? (fysiek)	☐1	☐2	☐3	☐4	☐5
d.	gepest door buitensluiten, negeren of roddelen? (relationeel)	☐1	☐2	☐3	☐4	☐5

Hoe vaak heeft deze leerling in de afgelopen maanden…

		niet	1 of 2 keer	2 of 3 keer per maand	ongeveer 1 keer per week	meerdere keren per week
a.	andere leerlingen gepest (algemeen)	☐1	☐2	☐3	☐4	☐5
b.	andere leerlingen gepest door belediging, uitschelden, of uitlachen? (verbaal)	☐1	☐2	☐3	☐4	☐5
c.	andere leerlingen gepest door spugen, slaan, schoppen of knijpen? (fysiek)	☐1	☐2	☐3	☐4	☐5
d.	andere leerlingen gepest door buitensluiten, negeren of roddelen?(relationeel)	☐1	☐2	☐3	☐4	☐5

Table S1. *Steps in the Model Fitting Procedure*

Steps	Model-fitting tests
	Mean Structure
1	Classroom differences (Same Classroom vs. Different Classrooms)
2	Sex differences (Boys vs. Girls)
	Variance Components
3	Classroom differences in all genetic parameters
	(a11 a21 a22 $_{SC}$ = a11 a21 a22 $_{DC}$)
4	Classroom differences in all common environmental parameters
	(c11 c21 c22 $_{SC}$ = c11 c21 c22 $_{DC}$)
5	Classroom differences in unique environmental covariation
	(*r* e1-e2 $_{SC}$ = *r* e1-e2 $_{DC}$)
6	Sex differences in all genetic parameters
	(a11 a21 a22 $_{boys}$ = a11 a21 a22 $_{girls}$)
7	Sex differences in all common environmental parameters and in unique environmental covariation
	(c11 c21 c22 & *r* e1-e2 $_{boys}$ = c11 c21 c22 & *r* e1-e2 $_{girls}$)

Note. SC = Same Classroom. DC = Different Classrooms. a = genetic influences. c = common environmental influences. e = unique environmental influences.

Table S2. *Twin Correlations for General Victimization and Perpetration*

	Within Traits				Cross-Traits	
	General Victimization		General Perpetration		General Victimization with General Perpetration	
	Same Class	Different Class	Same Class	Different Class	Same Class	Different Class
MZ boys	.83 (.78-.87)	.42 (.27-.58)	.86 (.83-.90)	.55 (.44-.67)	.60 (.53-.67)	.31 (.18-.43)
MZ girls	.86 (.81-.92)	.34 (.17-.51)	.89 (.85-.93)	.39 (.22-.56)	.56 (.46-.67)	.20 (.05-.35)
DZ boys	.62 (.53-.71)	.24 (.07-.41)	.55 (.45-.65)	.39 (.24-.54)	.37 (.26-.47)	.19 (.05-.33)
DZ girls	.71 (.61-.80)	.14 (-.10-.39)	.65 (.54-.77)	.38 (.16-.59)	.55 (.43-.68)	.19 (.02-.36)
DOS	.52 (.43-.62)	.14 (.00-.29)	.45 (.36-.55)	.21 (.07-.35)	.39 (.28-.50) - .29 (.17-.41)*	.17 (.04-.30) - .09 (-.06-.25)

Note. MZ = Monozygotic twins. DZ = Dizygotic twins. DOS = Dizygotic twins of Opposite Sex.

* Correlations are between the trait in twin 1 and the trait in twin 2. The first value is the correlation between the girls' victimization score and the boys' perpetration score. The second value is the correlation between the girls' perpetration score and the boys' victimization score.

Table S3. *Twin Correlations for Verbal Victimization and Perpetration*

	Within Traits				Cross-Traits	
	Verbal Victimization		Verbal Perpetration		Verbal Victimization with Verbal Perpetration	
	Same Class	Different Class	Same Class	Different Class	Same Class	Different Class
MZ boys	.83 (.79-.88)	.45 (.31-.59)	.81 (.76-.86)	.57 (.45-.69)	.54 (.46-.63)	.32 (.19-.45)
MZ girls	.87 (.81-.92)	.41 (.23-.59)	.86 (.81-.91)	.56 (.38-.73)	.60 (.50-.70)	.47 (.32-.63)
DZ boys	.65 (.57-.73)	.13 (-.03-.29)	.50 (.40-.60)	.26 (.11-.41)	.38 (.28-.47)	.15 (.01-.28)
DZ girls	.68 (.56-.80)	.27 (.06-.49)	.58 (45-.70)	.27 (.03-.52)	.42 (.27-.56)	.20 (.03-.37)
DOS	.50 (.40-.60)	.16 (.02-.30)	.55 (.45-.64)	.31 (.18-.44)	.30 (.19-.42) - .40 (.29-.52)*	.26 (.14-.39) - .09 (-.06-.25)

Note. See notes of Table S3.

Table S4. *Twin Correlations for Physical Victimization and Perpetration*

	Within Traits				Cross-Traits	
	Physical Victimization		Physical Perpetration		Physical Victimization with Physical Perpetration	
	Same Class	Different Class	Same Class	Different Class	Same Class	Different Class
MZ boys	.89 (.82-.96)	.51 (.29-.74)	.90 (.84-.96)	.51 (.31-.72)	.78 (.69-.87)	.51 (.34-.68)
MZ girls	.93 (.85-1.00)	.60 (.23-.98)	.87 (.76-.99)	.69 (.35-1.00)	.76 (.59-.93)	.65 (.32-.99)
DZ boys	.68 (.54-.82)	.32 (.04-.60)	.61 (.46-.76)	.18 (-.07-.43)	.51 (.36-.67)	.38 (.18-.57)
DZ girls	.67 (.41-.94)	.02 (-1.00-1.00)	.66 (.39-.93)	.39 (-.14-.92)	.63 (.41-.84)	.10 (-.74-.94)
DOS	.51 (.32-.69)	.02 (-.34-.38)	.40 (.19-.61)	.20 (-.06-.47)	.53 (.36-.70) - .22 (-.05-.48)*	.12 (-.16-.40) - .03 (-.33-.39)

Note. See notes of Table S3.

Table S5. *Twin Correlations for Relational Victimization and Perpetration*

	Within Traits				Cross-Traits	
	Relational Victimization		Relational Perpetration		Relational Victimization with Relational Perpetration	
	Same Class	Different Class	Same Class	Different Class	Same Class	Different Class
MZ boys	.79 (.72-.87)	.49 (.29-.69)	.84 (.79-.89)	.48 (.31-.66)	.54 (.42-.66)	.17 (-.02-.35)
MZ girls	.86 (.81-.90)	.31 (.13-.50)	.86 (.82.91)	.32 (.14-.51)	.52 (.43-.62)	.28 (.12-.45)
DZ boys	.67 (.57-.78)	.45 (.26-.63)	.58 (.45-.70)	.31 (.13-.49)	.48 (.37-.60)	.04 (-.15-.22)
DZ girls	.72 (.62-.82)	.08 (-.15-.32)	.65 (.54-.76)	.07 (-.16-.31)	.54 (.42-.66)	-.05 (-.23-.14)
DOS	.51 (.39-.63)	.08 (-.08-.24)	.51 (.40-.62)	.21 (.05-.36)	.38 (.24-.51) - .37 (.24-.50)*	.09 (-.07-.24) - -.08 (-.25-.10)

Note. See notes of Table S3.

Table S6. *Estimates (in %) for variation due to Additive genetic, Common environmental, and Unique environmental factors for all Types of Victimization and Perpetration and their Correlation, with the Rater-effects included*

	Perpetration								Victimization								Correlation			
	A		C		E		Rater		A		C		E		Rater		r_A		Proportion due to A[1]	
	Boys	Girls	Boys	Girls	Boys	Girls	Boys	Girls	Boys	Girls	Boys	Girls	Boys	Girls	Boys	Girls	Boys	Girls	Boys	Girls
General	53		7	9	14	12	26		37		5	8	17	15	40		.50		34	32
Verbal	60		2	7	20	15	18		40		5	9	17	14	38		.62		47	42
Physical	53		9	11	12	10	26		46		10	12	10	8	34		.86		52	48
Relational	43		4	5	16	15	37		32		9	10	17	16	43		.26		16	14

Note. The genetic parameters are constrained to be equal for boys and girls, so there is only one A estimate for each item for both traits. The rater effect is equal for boys and girls as well. [1] The proportion of the correlation between perpetration and victimization that is due to A differs between boys and girls, despite equal genetic parameters, because the phenotypic correlation (between perpetration and victimization) differed. The remaining part is due to environmental influences (both common and unique).

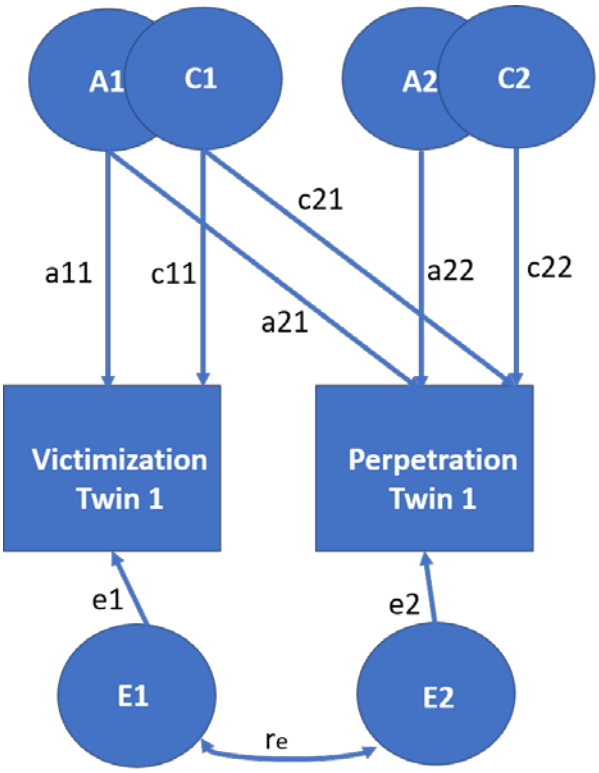

Figure S1. Within person specification for the ACE model used in this study. “A” represents the genetic, “C” the common environmental, and “E” the unique environmental influences. Rater effects are not shown to avoid clutter. “a11” represents the genetic influences on victimization, “a12” represents the genetic covariance between victimization and perpetration, and “a22” represents the unique genetic influences on perpetration after accounting for the shared genetic influences. “c11” represents the common environmental influences on victimization, “c12” represents the common environmental covariance between victimization and perpetration, and “c22” represents the common environmental influences on perpetration after accounting for the shared common environmental influences. “e1” represents the unique environmental influences (modeled as a residual) on victimization and “e2” the unique environmental influences (residual) on perpetration. “r_e” represents the unique environmental correlation between the residual of victimization and the residual of perpetration, and only this parameter for the unique environmental part of the model is estimated.
